# Similar genomic proportions of copy number variation within gray wolves and modern dog breeds inferred from whole genome sequencing

**DOI:** 10.1186/s12864-017-4318-x

**Published:** 2017-12-19

**Authors:** Aitor Serres-Armero, Inna S. Povolotskaya, Javier Quilez, Oscar Ramirez, Gabriel Santpere, Lukas F. K. Kuderna, Jessica Hernandez-Rodriguez, Marcos Fernandez-Callejo, Daniel Gomez-Sanchez, Adam H. Freedman, Zhenxin Fan, John Novembre, Arcadi Navarro, Adam Boyko, Robert Wayne, Carles Vilà, Belen Lorente-Galdos, Tomas Marques-Bonet

**Affiliations:** 1 IBE, Institut de Biologia Evolutiva (Universitat Pompeu Fabra/CSIC), Ciencies Experimentals i de la Salut, 08003 Barcelona, Spain; 2 CNAG-CRG, Centre for Genomic Regulation (CRG), The Barcelona Institute of Science and Technology (BIST), Barcelona, Spain; 3 Vetgenomics, 08193 Barcelona, Spain; 4 Department of Neuroscience, Yale School of Medicine, New Haven, CT USA; 5 UCLA, Department of Ecology and Evolutionary Biology, Los Angeles, CA 90095 USA; 6 Key Laboratory of Bioresources and Ecoenvironment (Ministry of Education), College of Life Sciences, Sichuan University, Chengdu, 610064 People’s Republic of China; 7 Institucio Catalana de Recerca i Estudis Avançats (ICREA), 08010 Barcelona, Catalonia Spain; 8 Cornell University, Department of Biological Statistics and Computational Biology, New York, NY 14853 USA; 9 Estación Biológica de Doñana EBD-CSIC, Department of Integrative Ecology, 41092 Sevilla, Spain

**Keywords:** Copy number variation, Dog genomics, Evolution, Domestication

## Abstract

**Background:**

Whole genome re-sequencing data from dogs and wolves are now commonly used to study how natural and artificial selection have shaped the patterns of genetic diversity. Single nucleotide polymorphisms, microsatellites and variants in mitochondrial DNA have been interrogated for links to specific phenotypes or signals of domestication. However, copy number variation (CNV), despite its increasingly recognized importance as a contributor to phenotypic diversity, has not been extensively explored in canids.

**Results:**

Here, we develop a new accurate probabilistic framework to create fine-scale genomic maps of segmental duplications (SDs), compare patterns of CNV across groups and investigate their role in the evolution of the domestic dog by using information from 34 canine genomes. Our analyses show that duplicated regions are enriched in genes and hence likely possess functional importance. We identify 86 loci with large CNV differences between dogs and wolves, enriched in genes responsible for sensory perception, immune response, metabolic processes, etc. In striking contrast to the observed loss of nucleotide diversity in domestic dogs following the population bottlenecks that occurred during domestication and breed creation, we find a similar proportion of CNV loci in dogs and wolves, suggesting that other dynamics are acting to particularly select for CNVs with potentially functional impacts.

**Conclusions:**

This work is the first comparison of genome wide CNV patterns in domestic and wild canids using whole-genome sequencing data and our findings contribute to study the impact of novel kinds of genetic changes on the evolution of the domestic dog.

**Electronic supplementary material:**

The online version of this article (doi:10.1186/s12864-017-4318-x) contains supplementary material, which is available to authorized users.

## Background

The dog (*Canis familiaris*) was domesticated from the gray wolf (*C. lupus*) [1–4] more than 10,000 years ago, although when and where domestication happened as well as the role of humans in the process have been focus of intense debate [5–10]. Beginning several hundred years ago, modern dog breeds were established as isolated gene pools, in parallel with strong artificial selection for specific physical and behavioral phenotypes favored by humans. A large number of dog breeds have been developed since then, which has resulted in a broad variety of traits and exceptional phenotypic variation [11].

Detecting and understanding the footprint that domestication left in the canine genome is an area of active research. To this end, genetic variation in dogs and wolves has been extensively studied using single nucleotide polymorphisms (SNPs) and microsatellites [12–16]. These studies have shown that nucleotide diversity is between 1.5 and 2 times lower in dogs than in wolves as a result of a 9 to 16-fold reduction in the effective population size associated with dog domestication [4, 17, 18]. Selective breeding further led to reduction in variation, longer linkage disequilibrium (LD) blocks and a lower number of haplotypes among purebred dogs compared to wolves and “village dogs”, which have not gone through the breeding process [15, 17, 19–22]. This reduction in diversity is striking in the light of the great phenotypic variation observed in modern dog breeds [12]. Several studies have focused on the identification of functional variants responsible for phenotypic changes associated with domestication [23] or contributing to phenotypic variation of the modern dog breeds [11, 19, 24–29].

Although CNV contributes to phenotypic differences and genetic diseases [28, 30–32], structural variation in multiple canine genomes has not been thoroughly interrogated yet genome-wide. Absolute copy number (CN) values in short genomic windows can be predicted computationally from whole genome sequencing experiments [33–40] and this approach has been used to study CNV patterns in many species. A number of studies have investigated CNV in dogs and wolves using experimental approaches, namely array comparative genomic hybridizations (aCGH) [30, 41–45] and intensity data from SNP genotyping arrays [46]. However, these techniques are limited to relatively low CN regions [47], produce CN values relative to the CN in the reference individual [48], have strong limitation in size of the detectable structural variants [49, 50] and only the parts of the genome in which probes have been placed can be interrogated [47].

In the present study, we aimed to investigate CNV regions in dogs and wolves. However, the analysis of the genome-wide patterns of segregating CNV across a set of individuals is a challenging task and requires precise estimates of the absolute CN of each CNV locus for each of the individual genomes. The accuracy of all the existing methods for absolute CN inference decreases rapidly as CN increases, and thus, nearly all of the studies of CNV diversity up to date are limited to biallelic loci with segregating alleles CN_1_ and CN_2_ per haplotype [40, 51, 52]. In addition, methods based on read depth only produce point estimates and do not provide confidence intervals, which are extremely important to distinguish between true CN variability and increased technical noise (especially for higher CN values) [53]. This is an important caveat considering that, as reported in humans, population differentiation in loci with a high number of copies might be an important contributor to phenotypic differences [40, 54, 55]. Here, we designed a new probabilistic framework of the read depth based approach for accurate absolute CN inference and CNV detection, which enabled us to perform a comprehensive genome-wide analysis of the patterns and dynamics of CNV loci across the entire range of CNs in a set of 34 canid genomes.

## Results

We analyzed a set of 34 sequenced individuals at a mean initial coverage of 16.8X [4, 56, 57]. Our dataset included 12 dogs (*C. familiaris*), 16 gray wolves (*C. lupus*), 2 red wolves (*C. rufus*), 3 coyotes (*C. latrans*) and 1 golden jackal (*C. aureus*) (Table 1) from diverse populations and breeds across Europe, America and Asia [57].

**Table 1:** Samples and sequencing coverage

Species	Sample	Abbreviation	HMM function	Raw coverage	Effective coverage	aCGH data	Dataset	Diversity analysis
Dog	Chinese indigenous dog	DogCI2	Training	9.83	–	No	Wang et al.	No
Dog	Dingo	din	Analysis	7.09	5.1	No	Freedman et al.	Yes
Dog	Basenji	mba	Analysis	11.8	8.49	Yes	Freedman et al.	Yes
Dog	Kerry Blue Terrier	ali	Analysis	21.28	15.32	No	Fan et al.	Yes
Dog	Boxer	bxr	Analysis	31.27	22.29	No	Fan et al.	Yes
Dog	English cocker	cec	Analysis	11.81	8.5	No	Fan et al.	Yes
Dog	Labrador retriever	dlr	Analysis	12.6	9.07	No	Fan et al.	Yes
Dog	Chinese crest	jcc	Analysis	19.04	13.71	No	Fan et al.	Yes
Dog	Standard poodle	osp	Analysis	12.91	9.29	No	Fan et al.	Yes
Dog	Belgium Malanois	DogBM	Analysis	10.11	7.57	No	Wang et al.	Yes
Dog	German shepherd	DogGS	Analysis	9.56	5.61	No	Wang et al.	Yes
Dog	Tibetan Mastiff	DogTM	Analysis	10.37	5.8	No	Wang et al.	Yes
Gray wolf	Wolf Russia	GW3	Training	11.1	–	No	Wang et al.	No
Gray wolf	Wolf China	chw	Analysis	17.94	12.91	Yes	Freedman et al.	Yes
Gray wolf	Wolf Croatia	crw	Analysis	9.73	6.94	No	Freedman et al.	Yes
Gray wolf	Israeli wolf	isw	Analysis	7.37	5.26	No	Freedman et al.	Yes
Gray wolf	Wolf Great Lakes	glw	Analysis	26.8	19.3	Yes	Fan et al.	Yes
Gray wolf	Wolf India	inw	Analysis	27.42	19.74	Yes	Fan et al.	Yes
Gray wolf	Wolf Iran	irw	Analysis	30.15	21.71	Yes	Fan et al.	Yes
Gray wolf	Wolf Italy	ita	Analysis	7.59	6.07	Yes	Fan et al.	Yes
Gray wolf	Wolf Mexico	mxa	Analysis	25.64	18.46	Yes	Fan et al.	Yes
Gray wolf	Wolf Mexico	mxb	Analysis	7.08	5.66	No	Fan et al.	No
Gray wolf	Wolf Portugal	ptw	Analysis	28.46	20.49	Yes	Fan et al.	Yes
Gray wolf	Wolf Spain	spw	Analysis	28.88	20.79	Yes	Fan et al.	Yes
Gray wolf	Wolf Yellowstone	ysa	Analysis	28.21	20.31	Yes	Fan et al.	Yes
Gray wolf	Wolf Yellowstone	ysb	Analysis	18.82	13.55	Yes	Fan et al.	No
Gray wolf	Wolf Yellowstone	ysc	Analysis	8.44	6.75	Yes	Fan et al.	No
Gray wolf	Wolf China	GW4	Analysis	9.61	6.75	No	Wang et al.	No
Coyote	Coyote California	cac	Training	26.87	19.35	No	Fan et al.	No
Coyote	Coyote Alabama	alc	Analysis	7.69	5.54	No	Fan et al.	No
Coyote	Coyote Midwest	mwc	Analysis	9.11	6.56	No	Fan et al.	No
Jackal	Golden Jackal Kenya	jaa	Analysis	27.47	19.78	Yes	Freedman et al.	No
Red wolf	Red wolf	rwa	Analysis	30.28	21.8	No	Fan et al.	No
Red wolf	Red wolf	rwb	Analysis	7.72	6.17	No	Fan et al.	No

Sequences were retrieved from previously published work from Fan et al. [57], Freedman et al. [4] and Wang et al. [56]. The raw coverage is calculated from the total number of reads before mapping and referred to the 2,413,045,422 bps of the prepared version of CanFam3.1. The effective coverage is calculated after removing poor-quality sequencing lanes and read ends. For 14 samples aCGH data from Ramirez et al. [43] were available. Coyote, jackal and red wolf samples were combined as a single group for the analyses

We generated individual genome-wide fine-scale CN profiles using a previously published method [33]. Further, we developed and applied a new probabilistic approach, which allowed us to overcome some of the limitations of the previous methods by estimating probabilities for each CN and broaden the analysis to include loci of high CN.

### Validation

We validated our computational predictions with the available aCGH data [43] for 14 of the samples that are common in both studies (Table 1). We compared “digital” log2ratios between the reference individual (“bxr”) and each of the other samples included in the aCGH study [43], which showed a high correlation with the aCGH log2ratios (mean correlation coefficient *R* = 0.77 ± 0.06, Additional file 1: Table S1). Additionally, 95.4 ± 3.3% of windows with sample specific CN gains relative to the reference individual (Boxer) have passed the validation threshold (See METHODS and Additional file 1: Table S1). Boxer specific duplications had a lower validation rate (69.3 ± 6.8%), most likely as a result of sequencing biases specific to this sample (Additional file 1: Figure S1).

### Genomic duplications

**Fig. 1:**
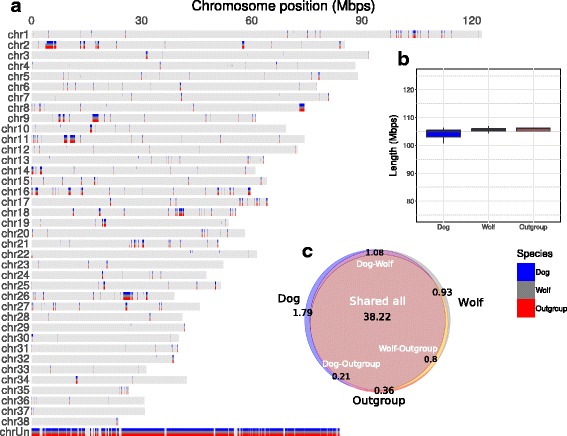
Landscape of canine segmental duplications. **a** Genome-wide map of canine SDs. Autosomes are represented by horizontal bars, and each mark represent a duplicated region identified in at least one sample of the group indicated. **b** Total length of genomic duplications identified per subspecies. **c** Venn diagram showing intersection of duplicated regions identified in dogs, gray wolves and canines in the outgroup (chrUn excluded)

Duplicated genomic regions spanned 114.05 Mbps (43.44 Mbps in autosomal chromosomes and 70.69 Mbps in unplaced scaffolds) or about 5% of the size of dog autosomes. Dogs have 111.82 Mbps of duplicated sequence, gray wolves 111.46 Mbps and related canids 109.74 Mbps. We found, that 79% of the genomic duplications were present in all the individuals (89.72 Mbps in total, 24.03 Mbps in chromosomes and 65.70 Mbps in unplaced scaffolds), 93.04 Mbps (~83%) were present in all the dogs and 95.53 Mbp (~86%) in all the wolves (Fig. 1a). Dogs and gray wolves showed the same average amount of duplicated sequence per individual (104.21 ± 1.89 and 105.54 ± 0.71 Mbps, respectively, Fig. 1b) and 38.22 Mbps were duplicated in at least one individual from each subspecies excluding unassembled scaffolds (Fig. 1c). The average length of duplicated segments did not depend on the sample coverage (Additional file 1: Figure S2).

We found that the set of genomic duplications detected in the 34 canine samples overlapped with 433 genes annotated in the CanFam3.1 dog genome assembly, an overlap significantly higher than the random expectation (randomization *p*-value = 0.0023) (Additional file 1: Figure S3). Moreover, we found a significant enrichment of duplicated genes involved in detection of chemical stimulus and G-protein coupled receptor signaling pathways, both with p-val < 10^−30^ (Additional file 1: Table S2). These two pathways are closely associated with the perception and transduction of smell and other sensory functions. We also detected a significant enrichment in the pathways of immunoglobulin production and phagocytosis recognition with a *p*-value of ~10^−6^. Many essential genes were duplicated in all of our samples, including major cytoskeleton components, a number of ribosomal genes/proteins, mitochondrion maintenance and ubiquitination enzymes or DNA repair mechanisms among many others.

We further looked at the private duplications, present in one subspecies and not in the other. We restricted subsequent analysis to include only 11 dogs and 11 wolves from distinct populations (Table 1, Additional file 1: Table S3), consequently these differences do not result from different sample sizes of dogs and gray wolves (see METHODS). The number of duplications that were unique to dogs (3.67 Mbps or ~3.29% of dog duplications) was substantially greater than for gray wolves (2.19 Mbps or ~1.97% of gray wolf duplications) and they mainly corresponded to events in single individuals (Additional file 1: Figure S4) and none of the private duplications was shared by more than 7 individuals. These private duplications were also significantly enriched in genes for both dogs (randomization *p*-value = 0.0075) and wolves (randomization *p*-value = 0.003) with genes involved in iron homeostasis and elastin catabolism overrepresented in dogs, and genes involved in arginine transport overrepresented in wolves (Additional file 1: Table S4).

### Genomic proportion of CNV

**Fig. 2:**
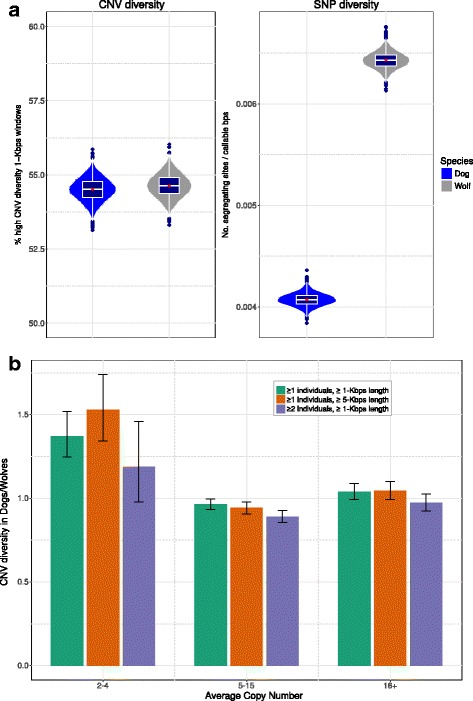
Proportion of genomic CNV and SNP diversity in dogs and gray wolves. **a** Boxplots indicate the observed values of overall genomic proportion of CNVs and SNP diversity in dogs and gray wolves. Violin plots correspond to 5000 bootstrap values. **b** Ratios of the level of CNVs in dogs to the level of CNVs in wolves for low (2–4), medium (5–15) and high (16+) copy number categories. In green all the regions are taken into account, in orange short regions (less than 5 consecutive windows) of variable CN are filtered and in purple singletons are filtered

Our CN calls allowed us to identify windows with segregating CN alleles within populations. We assessed whether the proportion of the genome classified as CNV was reduced in the dog lineage relative to the gray wolf, as has happened for nucleotide diversity (Fig. 2a) reflecting domestication and breed creation bottlenecks. As an overall measure of the fraction of the genome with segregating CNV in either subspecies, we used the number of 1-Kbps windows for which at least two individuals presented non-overlapping CN intervals (further referred to as variable windows specifically or CN variability globally) divided by the total number of 1-Kbp windows called inside duplications, taken as the most likely substrate for CNVs [58, 59]. In striking contrast to the 1.6-fold reduction in single nucleotide diversity in our dataset of dogs (in accordance with estimates of 1.5 to 2-fold reduction reported previously [4, 17, 18], see Additional file 1: Tables S4 and S5), we found similar proportion of the duplicated genome space with CNVs in the two canine subspecies (54.5% and 54.6% variable windows per total number of duplicated windows in dogs and wolves respectively) (Fig. 2a, Additional file 1: Table S6). Among all variable windows in dogs, 78.8% are also variable in wolves, while for wolves this proportion is slightly higher (80.9%). Most of these regions represent in principle, variability originated before the lineage split, whereas those regions not shared (21.2% and 19.1% respectively), represent subspecies-specific variability, which could potentially contribute to functional differences between the two subspecies (Additional file 1: Figure S5). Alternatively, these regions may represent independent inheritance of CNVs from a common ancestor.

We sought then to eliminate the possibility that artifacts of our CN calling algorithm might influence our estimates. Given the known differences in accuracy of depth of coverage methods for different CN magnitudes, we first divided all genomic duplications into three categories according to their corresponding CN. Specifically, these categories included duplications of low CN (mean CN across all windows in all duplicated individuals between 2 and 4), medium CN (mean CN between 4 and 15) and high CN (mean CN larger than 15). We then calculated variability levels for each of the categories separately (Additional file 1: Table S6). Surprisingly, the proportion of CNV windows within genomic duplications with low CN (2–4), is even higher in dogs than in wolves (28% and 20% respectively). In this category we assessed the quality of the calls of variable windows for each pair of samples with a two-way aCGH comparison. We required that the absolute value: ${\mathit{\log}}_2\frac{CN_{Sample1}}{CN_{Sample2}}={aCGH}_{Sample1}-{aCGH}_{Sample2}$ exceeds the cutoff of *aCGH*_*CUTOFF*_ = ±3*σ_*aCGH*_ *(CR)* (see METHODS for details) for all the windows with predicted CN differences between the two samples, when one of the samples was not predicted to be duplicated. We thus validated 89% of windows per sample for relative losses and 88% per sample for relative gains (median values, Additional file 1: Table S7).

To further investigate if our measure of CN variability is affected by singletons, we repeated the analysis requiring a minimum of two individuals to be called with a different CN. Even so, dogs and wolves presented similar genomic proportions of CNVs and the value in the low CN category is still slightly higher for dogs (Fig. 2b, Additional file 1: Table S6). Finally, we tested whether the similar levels of genomic variation are not driven by hyper variable duplication breakpoints [60] and are not a result of inaccurate calls of short variable regions. To do so we required for CN regions to be comprised of a minimum of 5 consecutive windows which are identified as variant within the population, and still found overall similar genomic proportion of CNVs comparing dogs and wolves (Fig. 2b, Additional file 1: Table S6).

Variable duplicated genomic segments, defined as 1-Kbps windows for which there were at least two individuals with non-overlapping CN intervals, are enriched in genes in the low and medium CN categories for both lineages (dogs: p_CN2–4_ = 0.018 and p_CN5–15_ = 0.023; wolves: and p_CN2–4_ = 0.014 and p_CN5–15_ = 0.053) and many of these genes are involved in both innate immunity (6 genes related to phagocytosis recognition) and adaptive immunity (15 genes involved in immunoglobulin production and MHC maturation). A striking enrichment was found in the pathway of DNA recombination and the most significant signal belonged again to olfactory receptor activity (Additional file 1: Table S8).

**Fig. 3:**
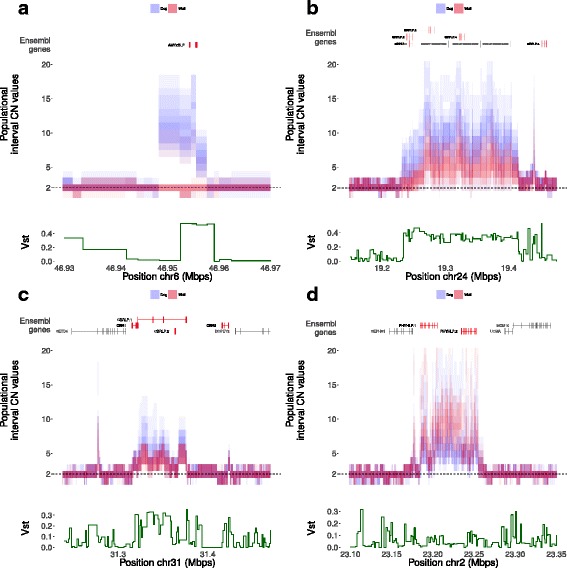
Genes in high V_ST_ between dogs and wolves. (**a**) AMY2B (**b**) SIRP (**c**) CBR1 (**d**) PHYH

We further looked for genes which show a high degree of CN differentiation between the two subspecies based on the V_ST_ statistic. We recover a number of genic CNVs previously reported to be associated with the dog specific phenotypes. Among these genes is the paralogue to the canine alpha-2B-amylase gene (*AMY2B*), which catalyzes the first step in the digestion of dietary starch and glycogen (Fig. 3a and Additional file 1: Table S9). Another case of CN expansion in dogs is a 150-Kbps duplication in chromosome 24 [16, 42]. This duplication spans three members of the signal-regulatory protein (SIRP) gene family, which mediate immune-cell regulation [61] (Fig. 3b and Additional file 1: Table S9). Similarly, the *CBR1* gene (Fig. 3c), coding for a carbonyl reductase enzyme involved in the degradation of both environmental and biologically synthesized quinones, lies within a region duplicated in most samples with some dog samples having a higher number of copies (Additional file 1: Table S9).

## Discussion

Inferring absolute CN values from sequencing read depth and determining gains in the number of copies is not trivial. Among computational methods, read depth based approaches are the most accurate [62]. Here, we develop an accurate probabilistic expansion of the sequencing read depth based method to call CN genome wide and use this method to produce fine-scale maps of genomic duplications and CNV regions in dogs, gray wolves and the more basal coyote and golden jackal lineages. The novelty of our approach relies in the population-wide Bayesian probabilistic method to CN estimation, hence allowing us to reliably compare CN values across groups of genomes.

The CNV and duplication maps that we present in this study greatly improve on the landscape of structural variation in the canine genome. We analyzed a set of dogs from different breeds, but we additionally included a wide range of gray wolf samples from a broad geographic distribution and several individuals from other wild canine species. All samples were previously sequenced [4, 56, 57] by next generation sequencing and we utilized a computational read depth approach to estimate fine-scale CN for each individual.

The main objective of our study was to investigate whether the proportion of the genome with CNV regions is reduced in dog compared to gray wolf genomes, which would indicate a reduction of CN polymorphisms similar to the expectations based on SNP diversity and the inferred bottlenecks. To do that, precise estimates of the absolute CN of each CNV locus for each of the individual genomes and a probability associated to them are required. We applied an HMM prediction, local multi-sample re-genotyping and created accurate interval estimates of absolute CNs. We further validated our calling method with an available experimental dataset and found that the accuracy of the method is comparable and in some cases slightly superior to the accuracy of previous methods for copy gain predictions. Accuracy of the calls varies across samples but is not dependent on coverage depth (Additional file 1: Figure S6), as expected since the uncertainties associated with coverage are taken into account by HMM predictions and multi-sample re-genotyping.

Our fine-scale duplication maps indicate that dog genomes present similar genomic proportion of CNV compared to those of gray wolves (Fig. 2a). Nucleotide diversity in dogs compared to their canine ancestors has been reduced genome-wide, as reported previously [4, 17, 18] and supported by our data in an extended set of samples (Fig. 2a, Additional file 1: Table S5 and Additional file 1: Table S6). This reduction in nucleotide diversity has been attributed to the population bottlenecks and inbreeding that dogs have suffered as a result of domestication and the creation of dog breeds [13, 20, 63]. With regard to duplications, we observe that ~80% of the CNV sites are mostly shared by both subspecies. It is notable that still ~20% of these genomic CNV regions are not shared, and they might then contribute to the phenotypic plasticity observed in modern dog breeds or represent different sampling of CNV regions from a common ancestor.

We explicitly addressed potential biases that could affect the calculation of the proportion of CNVs. First, the proportions are maintained when considering a high confidence subset of regions, for which at least two individuals are called with a different CN. As for each individual we require the cumulative probability of the CN interval to reach at least 0.99, the probability that two distinct individuals would be called incorrectly is lower than 10^−4^. Second, accuracy of duplication calls increases with the length of the duplicated region [33] and the same is true with the accuracy for the calls of variability. After exclusion of all short variable segments, the resemblance between CN variation levels in the two subspecies is still maintained and the relative genomic CNV proportion in the low CN category even increases in dogs (Fig. 2b).

Finally, perhaps the greatest challenge in our estimates of the genomic proportion of variable duplications, is the fact that the total length of duplications represented in the dog assembly that are unique to dog samples might be either collapsed or misrepresented. To determine the extent of this problem in our estimates, we used the duplications identified in the genome with the whole-genome assembly comparison (WGAC) [42] to count each duplication detected in each subspecies. After correcting for duplications annotated in CanFam2, we found a slight increase in the proportion of CNVs observed in the dog compared to the wolf, although the final magnitudes were reduced 15–20% in both subspecies (Additional file 1: Figure S7). It is worth mentioning that our approach is based on counting the proportion of variable windows or equivalently the proportion of total length of variable duplications in the entire duplicated space and therefore it is just an estimation of the actual duplication units. For a more accurate assessment, better resolution of duplication events and breakpoints is required, which could be achieved by whole genome reconstruction based on long read sequencing technologies.

Altogether, similar levels of CNV load in dogs and wolves are extremely unlikely to be explained by an artifact or a bias alone. A key question is then why CN variability is not as reduced in dogs compared to single-nucleotide variation. Below, we considered each of the forces driving mutation-selection-migration equilibrium separately.

There are two scenarios in which selection might increase CNV levels in dogs above those expected given their demographic history. First, the maintenance of relatively high CNV levels in dogs is consistent with diversifying selection among different canine populations if regions of CNV are strongly functional. However, if that is the case, selected functional variants should show high frequency in breeds sharing a trait under selection and be at low frequency or absent in other dogs, resulting in a high overall proportion of genomic CNV. Although, this idea is difficult to test with the current dataset due to the limited number of samples per dog breed, data with aCGH suggest that most of the CNV found in dogs are not shared within breeds but across individuals of different breeds [30]. However, this data does not eliminate across breed variability in high CNs, which would not be detected given the lower dynamic range for such values in aCGH. Alternatively, domestication has relaxed selective pressure on dogs [64] and the consequences of this relaxation can be seen in differences in coding sequence variation [65]. Then, if CNV is generally slightly deleterious, the reduced efficiency of natural selection in small populations during the domestication bottleneck might affect CNVs differently than general SNP diversity especially if the distribution of selective effects is biased toward a greater frequency of neutral or nearly neutral variants in CNVs.

The CNV mutational landscape might also be altered in the canine lineage. Notably, the recombination hotspot gene *PRDM9* gene was pseudogenized in the dog genome. This gene is involved in recombination and novel CNV formation in primate and rodent lineages [66, 67]. Its absence in the dog genome might imply different conditions for CNV formation in the canine lineage. The genomes of closely related domestic cat, panda and ferret all carry a functional copy of *PRDM9*. Interestingly, a region with *RPA3*, one of the genes which binds and stabilizes single-stranded DNA during DNA replication and plays a role in double-strand break repair via homologous recombination, is duplicated in all canid genomes in our study and is variable in dogs. Given 80% of CNVs are shared between the two subspecies, many of them likely originated before the two lineages split, but it could also indicate recurrent duplication events happening at hotspots. However, great uncertainty exists about the overall mutation rate of SNPs [22, 68] and CNV in canines and even less is known about the variation of this rate between dogs and wild canids.

Finally, a reduction in our estimates of CNV relative to SNP diversity also could have been accomplished by reducing the number of genotypes [69, 70] that are segregating in dogs. CNV loci carry on average more alleles than SNP loci, which normally carry just two [71–73]. Although the dynamics of the loss of the number of alleles might be similar between two types of variation, the levels of variability in case of CN will be affected less [35, 73]. The number of alleles per loci is higher for high CN regions [72] and thus, even with a significant reduction of the number of alleles per locus, the level of variability of those high CN loci will not be reduced to the same degree. This effect might underlie the dynamics of our median and high CN categories. Remarkably, duplications with relatively low mean CN are consistently more variable in dogs than in wolves. These low CN duplications are significantly enriched in genes and some have subspecies specific variants, suggesting to a certain extent they might be novel and contribute to functional changes that have occurred after the lineages split.

Regardless of the proposed scenarios, some of the CNV loci with a high degree of variability in dogs or wolves, and specifically gene expansions in CN in the dog lineage, might affect phenotypic differences between subspecies given that 20% are unique to one of these subspecies. A good example is the unique amplification of the amylase gene CN in all dogs, as opposed to the single-copy number in almost all gray wolves, which has been linked to a starch-rich diet in dogs [23] (Fig. 3a). Another example is a highly variable tandem duplication of the *PHYH* gene [42], which in humans is linked to Refsum disease, with multiple epiphyseal dysplasia among variable features [74] (Fig. 3d). In addition, homozygous *PHYH* knockout mice exhibit slightly reduced tibia length [75]. We also detected a remarkable enrichment in the levels of SDs and CNVs in the pathways of immunoglobulin production and phagocytosis recognition, as a CNV region comprising the cluster of *SIRP* genes (Fig. 3b), which are involved in the adaptive immune system [61]. The levels of immunoglobulin A have been shown to vary greatly across dog breeds [76] but, to our knowledge, copy number has never been studied as a possible cause for this variation. An example of natural and artificial selection acting in opposite directions might be the widespread duplication upstream of the *KITLG* gene, which is linked to the increased risk for squamous cell carcinoma in black standard poodles [77]. *KITLG* locus has been shown to be under strong selective pressure in dogs [19] and a number of other species [78–80]. Interestingly, in humans and stickleback fish this locus is associated with variation in skin pigmentation [81, 82] and therefore possibly also plays a role in coat color and patterning in dogs. Thus, high frequency of this duplication might be explained by artificial selection favoring coat color traits preferred by humans, despite its negative impact on overall fitness.

## Conclusions

We present the first genome-wide assessment of CNV landscape in canids based on CN maps generated from high-coverage whole genome sequencing data. The novelty of this study resides in its focus on structural genome variation, which has not been as extensively explored as single-nucleotide variation in canids [4, 17, 19–22]. Additionally, we present a novel method for the application to the whole-genome sequencing read depth data to predict absolute genomic CN under a probabilistic framework. We find that the proportion of genome-wide CNVs in dogs and wolves has been maintained at similar levels in contrast to the decline of nucleotide variation seen in dogs. This result could reflect diversifying selection among dog breeds and populations if CNV are generally functional as with *AMY2B* [43]. The enrichment of genes in CNV regions further supports this assertion. Furthermore, we identify genes with divergent CN variation in dogs and gray wolves, which might have contributed to phenotypic and behavioral differences between the two subspecies. Determining the functional importance of CNV and amount of dog breed specific CNVs should be a focus of future studies.

## Methods

### Samples and sequencing data

We use sequence data from a panel of 22 canids including 6 dogs, 13 wolves and 3 coyotes sequenced previously [57]. Further, we included the genomes for another 12 canids recently published [4, 56], provided that they had a raw coverage greater than 5X (see below). Altogether, our final dataset comprised 12 dogs, 16 gray wolves, 2 red wolves, 3 coyotes and 1 golden jackal (Table 1) at a mean initial coverage of 16.8X [4, 56, 57]. Each dog sample was from a different so-called modern dog breed with the exception of the Dingo, Basenji and Chinese indigenous dog, which are typically regarded as old lineages. The wolves were sampled from a broad geographic distribution and included a family trio (male, female and offspring) from Yellowstone. For the subsequent analyses we considered the red wolves, coyotes and the jackal samples as a single group (referred to as “outgroup”).

### Pipeline for calling copy number from sequencing data

**Fig. 4:**
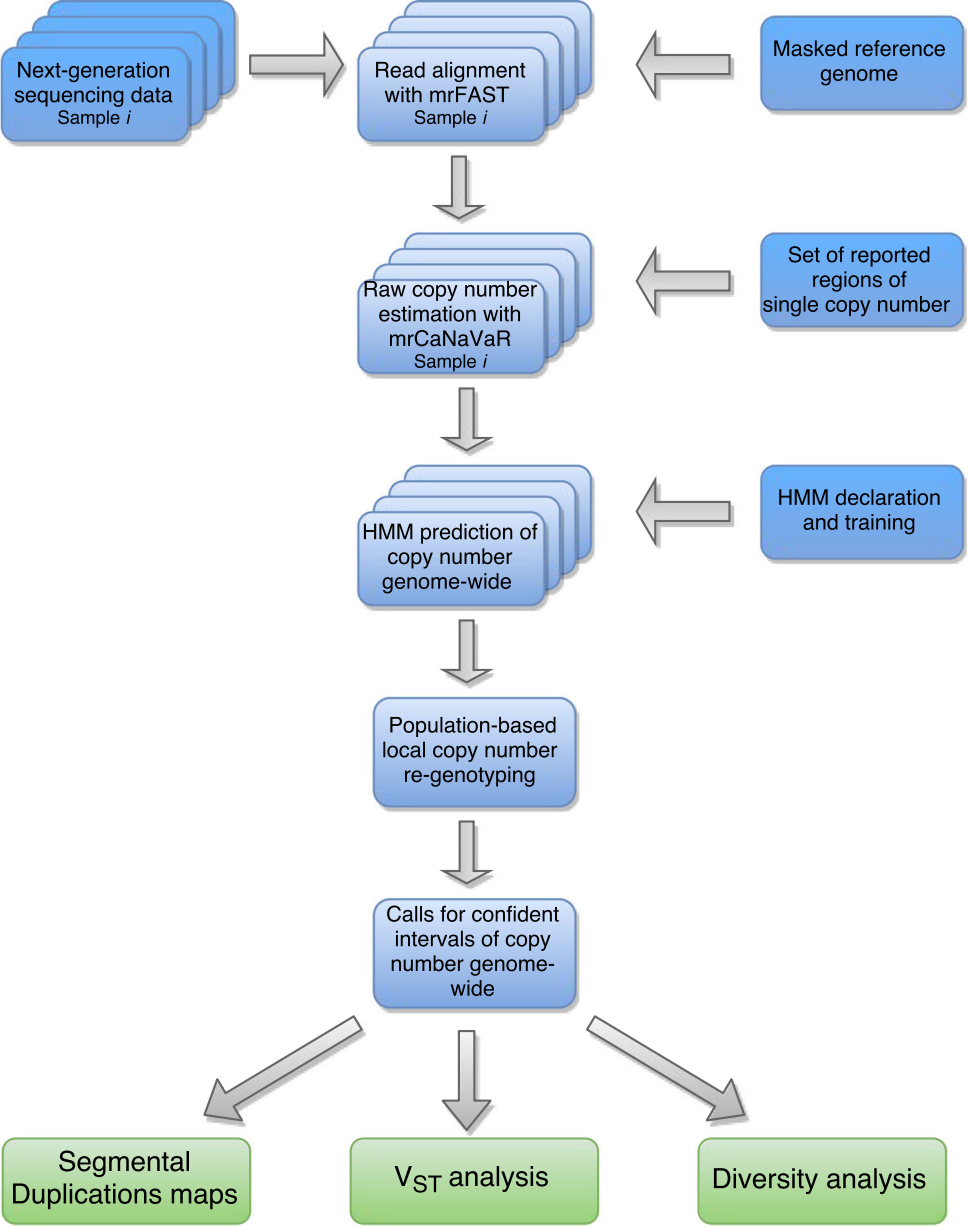
Pipeline for absolute copy number calling

We extended a read depth based approach for detection of SDs with a HMM for CN prediction from raw sequencing read depth and incorporated it to the pipeline for calling CN and CNV regions genome-wide (see Fig. 4 for the pipeline overview). To create raw, continuous, genome-wide CN predictions we applied a previously described [33] approach, which consists of the following steps:

(i) Masking of over-represented kmers in the assembly. In addition to the repeats already masked in the UCSC Genome Browser [83] version of CanFam3.1 with RepeatMasker [84] and Tandem Repeat Finder [85], we sought to identify and mask potential hidden repeats in CanFam3.1. In order to do so, chromosomes were partitioned into 36-bps kmers (with adjacent kmers overlapping 5 bps) and the resulting kmers were mapped against СanFam3.1 using mrsFast [86]. Then we masked positions in the assembly mapped by kmers with more than 20 placements in the genome, resulting in 6,910,707 bps additionally masked compared to the original CanFam3.1.

(ii) Mapping 36-bps reads against the assembly. Illumina reads from each individual were split into 36-bps portions (positions 10–45 and 46–81 of the original reads in order to exclude the lower-quality ends of the reads) and mapped to the prepared version of CanFam3.1 using mrFast [86].

(iii) Read depth calculation in 1-Kbps non-overlapping windows of non-repetitive sequence. To avoid edge problems with masked regions, which would underestimate the CN, the 36 bps flanking the masked regions were masked as well (referred to as 36-bps padding onwards). We then calculated the read depth in 1-Kbps non-overlapping windows of non-masked sequence.

(iv) GC-corrected absolute CN estimation from read depth. Read depth values in 1-Kbps windows were then corrected for GC bias using a set of diploid control regions. Control regions were defined as a set of diploid windows totally included in autosomal regions that had not been reported as a CNV in previous studies [4, 41, 42, 87]. These studies were based on CanFam2 and we lifted over the final set of control regions to CanFam3.1. Finally, we removed gaps (plus a 36-bps padding at the start and end of the gap) from the control regions in CanFam3.1. Altogether, this resulted in 21,260 control regions (mean = 94.9 Kbps) with a total size of 2,017,239,131 bps (83.7% of CanFam3.1) and the majority being smaller than 500 Kbps. Of the total 1,151,822 1-Kbps windows 998,077 (86.7%) and 153,745 (13.3%) were control and non-control, respectively.

(v) Raw CN estimation. Finally, we determined CN in the 1-Kbps windows of non-repetitive sequence as the read depth of each window divided the mean read depth in the control regions set.

We noted that this setup is equivalent to a Hidden Markov Process, where hidden states correspond to the true integral CN of the genomic region, emission symbols correspond to CN estimates based on read depth, emission probabilities are drawn from the corresponding read depth distributions and transitions between states correspond to genomic changes of the CN in the adjunct genomic regions. We thus applied this HMM approach to estimate the probabilities of each CN for 1 Kbp windows. We declared HMM states as a set of all possible CN for low CN *(*from *CN = 0 to CN = 20)* and their corresponding emission distributions as normal distributions with the mean corresponding to the CN, μ_N_ *= N,* and standard deviation derived from standard deviation in control regions *(CR, CN = 2)* as ${\sigma}_N=\sqrt{0.5N}\times {\sigma}_{CR}.$ For high CNs we declared states as an interval of CNs *(CN21–100, CN101–1000)* and modeled their emission distribution as a mixture of normal distributions with weights proportional to the estimated frequencies of each CN. We trained the transition matrix of this HMM with the Baum–Welch algorithm coded in the Pomegranate Python package [88] until convergence. We trained the HMM separately for dogs and wolves, using continuous CN predictions from read depth for one of the samples as observations (see Table 1). We excluded samples used for training from further analysis.

As samples differ greatly in coverage, which in turn leads to differences in standard deviations in the control regions, we redefined the HMM for each individual separately, so the emission distributions would resemble sample specific standard deviation of the read depth in regions of *CN = 2*. The transition matrix is, on the contrary, subspecies specific and does not depend on the sequencing quality. We predict probabilities of each of the declared states at each point by using the forward-reverse algorithm coded in the Pomegranate Python Package. For each individual we thus predict probabilities of each CN at each 1-Kbp window. Individual read depth based predictions of CN are very noisy and in order to improve them we additionally performed local population-based re-genotyping. For a particular observation of read depth derived raw CN *cn = x*, we use Bayes’ theorem to estimate the probability to draw this value from each of the distributions corresponding to CN states:

$$ p\left( CN=N| cn\in \left[x+ dx\right]\right)=\frac{p\left( cn\in \left[x+ dx\right]| CN=N\right)\times p\left( CN=N\right)}{p\left( cn\in \left[x+ dx\right]\right)}=\frac{PDF\left( cn,N,\sqrt{0.5N}{\sigma}_{CR}\right)\times dx\times p(N)}{\sum \limits_{CN} PDF\left( cn,N,\sqrt{0.5N}{\sigma}_{CR}\right)\times dx}=\frac{PDF\left( cn,N,\sqrt{0.5N}{\sigma}_{CR}\right)}{\sum \limits_{CN} PDF\left( cn,N,\sqrt{0.5N}{\sigma}_{CR}\right)}\times p(N); $$

where *p(N)* is the expected probability to observe *CN = N* in the data, the only variable which could be tuned locally. For every 1-Kbp window and each possible state of *CN = N*, we calculated its average probability across all the individuals in 5 consecutive windows, centered at the window of interest, and used this mean probability as a prior for the expected probability *p(N)* of *CN = N* in the data.

For a fraction of 1-Kbp windows (~2.5% inside duplications, ~51% genome wide) we can call the underlying CN with high confidence *(p > 0.99)* as a unique integer value. But for complex regions of high CN which are variable across individuals, the probability of each CN is low *(p < 0.99)*. For such windows we consider confidence intervals of the underlying CN. To do so, for each window, we order CN states according to their probability after population based local re-genotyping, and add them to the interval one by one, until their cumulative probability reaches *p = 0.99* threshold. We further call underlying CN of the window to belong to this interval. We thus could assess if for a particular window any two individuals have the same CN (if we confidently call them with the exact value), different (if we confidently call them to belong to non-overlapping CN intervals), or unresolved (if we call the individuals to belong to overlapping intervals).

We defined duplicated regions as regions of the genome, which harbor at least 5 consecutive windows, which we confidently call as CN > = 3 in at least one of the individual canine genomes. The collection of all such regions we call duplication track, and perform all further analyses only for windows which belong to this track.

### aCGH data and validation of the method

For 14 of the samples (1 dog, 1 jackal and 11 gray wolves and 1 red wolf) in which we predicted fine scale confidence CN values, aCGH data assays were available [43] (Table 1). This aCGH chip contains 598,733 probes which target, with a higher density, previously reported regions in the canine genome harboring structural variation [87]. In this study a Boxer sample was used as a reference in the array and we sequenced the same individual in the present study (bxr). Because the aCGH data was based on CanFam2 we generated the 1-Kbps CN predictions based on this version of the dog genome reference assembly and called confidence CN intervals for these 14 samples in the described fashion.

We performed quality control of aCGH experiments by assessing density function of aCGH probes for each individual (Additional file 1: Figure S8). The standard deviation for sample ysc was 2.5 times higher than for the rest of the samples, and we thus excluded ysc from subsequent aCGH validation analysis. We than calculated a threshold to separate true aCGH signals corresponding to gains and losses from diploid noise. To do so, we defined true CN = 2 windows as the intersection between regions which were previously experimentally identified as diploid [4, 41, 42, 87] and the regions which we confidently called as CN = 2 (probability greater than 0.99). As the aCGH chip was designed to target duplications and CNV regions previously reported in the canine genome, genome-wide 1-Kbp windows may be not covered uniformly with aCGH probes or covered at all, so we restricted our analysis only to the windows which harbor at least 2 different aCGH probes. We plotted the distribution of median aCGH signals for Boxer sample in these subset of windows (*n = 1452*), and used a cutoff for aCGH signal *CUTOFF = aCGH*_*MEAN*_ (CN = 2) ± 3*aCGH_*SD*_ *(CN = 2) = ±0.20* to discriminate between true gains and losses from false ones.

To validate our calls, we assessed if the difference in the CN which we predict computationally is confirmed by aCGH values. For each individual separately, we detected windows inside SDs, which we computationally predicted to be of a different CN than the reference Boxer sample. This difference could be a duplication compared to Boxer, if the sample CN is predicted to be higher than in Boxer, or a deletion compared to Boxer, if sample CN is lower than Boxer’s. We assessed the accuracy in detecting duplications and deletions separately, and calculated it as percentage of windows, which we predict to be CN different from Boxer, which have median aCGH above or below the *CUTOFF = ±0.2* respectively.

### Diversity analysis

Our probabilistic method has enabled us to analyze for the first time the fraction of CNV genome-wide and compare it to SNP diversity. To avoid sample sizes biases between dogs (*n* = 11) and gray wolves (*n* = 17), we matched the number of individuals from either subspecies by selecting a subset of 11 gray wolves based on various criteria (Additional file 1: Table S3); the selection of samples also ensured that only one gray wolf from each population was used.

### SNP calling and overall SNP diversity

After mapping sequencing reads to the canine genome with BWA [89], we used the CallableLoci tool of GATK [90], with default parameters, to determine areas of the genome that could be considered callable in each of the samples used in the analysis of CNV. We then defined the “callable genome” as the intersection of the callable regions across all the individuals. In addition, we subtracted from the callable genome the X chromosome and mitochondrial DNA, those regions that were masked in the version of the dog genome assembly used here (see above) and 1-Kbps windows with CN exceeding the sample-specific cutoff in at least one sample (Additional file 1: Table S5). After indel realignment we used the UnifiedGenotyper and VariantFiltration tools of GATK [90], with filtering parameters suggested when Variant Quality Score Recalibration (VQSR) is not available [91], to call SNP variants in the total of 11 dogs and 11 gray wolves used in the analysis of the genomic CNV proportion. For this analysis, however, we only retained those variants within the final callable genome (Additional file 1: Table S5). We then split SNPs into those seen in either dog or gray wolf samples and calculated, as a measure of overall SNP diversity, the number of segregating sites in either subspecies divided by the number of bps in the final callable, allowing for zero or two missing alleles (Additional file 1: Table S5). We also calculated the number of segregating sites per bps of callable using the subset of 8 dogs and 8 gray wolves with raw coverage >7X. We observed that the callable genome was greatly reduced by including those samples with a lower raw coverage (Additional file 1: Table S5). We therefore also performed the SNP calling and calculated SNP diversity in the subset of 8 dogs and 8 gray wolves with sequencing raw coverage >7X (see Table 1). We generated bootstrap values for the observed overall SNP diversity as follows: (i) partition the callable genome into intervals of 1 Mbps (*I*); (ii) random sampling with replacement of *I* intervals and re-calculated the number of segregating sites divided by the length of the callable genome.

### Genomic fraction of CNVs

Within dogs and gray wolves separately, we identify CNV windows as windows for which there are at least two individuals with non-overlapping predicted CN intervals. We measure variability within subspecies as percentage of variable windows among all the windows inside duplicated regions. In either subspecies we obtained an overall measure of CN variability as follows: (i) subset 1-Kbps windows which lie inside duplicated regions of a given subspecies (*N*); (ii) from those subset 1-Kbp windows which are variable in a given subspecies; (iii) generated bootstrap values by randomly sampling with replacement *N* windows and re-calculating CN variability, for a total of 5000 times.

To assess the patterns of variable CN across different CN values, we divided all the duplications into the CN bins. To each 1-Kbp window we assign a value, which is average of median points of CN intervals across individuals within subspecies. We further created bins of absolute CNs in such a way, that each bin contains at least 5% of the total number of duplicated windows: low CN (mean CN = 2–4), medium CN (mean CN = 5–15) and high CN (mean CN > 15). We classified all the windows to the bins and assessed the proportion of variable windows in each of them separately for dogs and wolves. To control for the high levels of noise in individual CN predictions we assessed variability for regions comprising at least 5 consecutive variable windows. As a separate control, we excluded singletons from the variability calls and required at least 2 individuals to belong to each of the non-overlapping CN intervals (Fig. 2, Additional file 1: Table S6).

### Genes overlapping with genomic duplications and enrichment analysis

We downloaded the 29,884 Ensembl gene models available for CanFam3.1 from the UCSC Genome Browser [83]. Additionally, we considered as of higher confidence those transcripts, 26,748 genes (89.51%), comprising at least one exon present in the xenoRef set of positions syntenic to exons in other species (*n* = 2,381,071), which was downloaded from the UCSC Genome Browser [83]. These transcripts were converted back to the gene coordinates and only the total of *N* = 20,328 genes in autosomes were considered for further analysis. For the gene enrichment tests we only selected genes which were entirely covered by duplications. We estimated the gene enrichment associated *p*-values by the bootstrap. We performed 10,000 repetitions of shuffling duplications coordinates, while keeping their true size and avoiding placing smaller duplications (<100 Kb) on gaps in order to generate an empirical distribution of the expected overlap between genes and SDs. The empirical p-value of the true observed value was calculated by dividing the rank of the true observation by the total number of permutations. The enrichment analysis was performed using the elimination algorithm of the TopGO R package [92], which scores GO terms hierarchically and subtracts specific, significant terms from the more global ones to avoid an overrepresentation of the latter. This conditions the results of the recursive tests on the topology of the gene ontology tree and reduces the effect of multiple testing to a level where no further conventional correction is required [93]. Instead, we refined our result set with the browser tool REVIGO [94], which implements semantic search algorithms in order to merge closely related GO terms and extract the most significant relations between them.

### Analysis of CNV differentiation between dogs and gray wolves

In every 1-Kbps window we used CN predictions in dog and gray wolf samples to calculate the V_ST_ statistic [51] between the two subspecies. The V_ST_ statistic is a variation of the F_ST_ [95] to measure between-populations differentiation in CNV regions:(*V*_T_ - V_S_)/*V*_T_ where *V*_T_ is the variance in the CN midpoints of all subspecies together, and V_S_ is the weighted average of the variance in CN midpoints for each subspecies separately. For consistency with the analysis of CNV and SNP diversity we calculated V_ST_ values between the same subsets of 11 dogs and 11 gray wolves (Additional file 1: Table S3). We looked for genes with median V_ST_ > 0.15 between dogs and wolves, which corresponds to the windows with the top 10% of V_ST_ values. We focused on the genes with more than 3 copies in dogs while less than 3 copies in wolves (Additional file 1: Table S9).

## Additional file

Additional file 1: Collection of all supplementary figures and tables. (DOCX 721 kb)
