# Supplementary material for: Similar genomic proportions of copy number variation within gray wolves and modern dog breeds inferred from whole genome sequencing
Source: BMC Genomics. 2017 Dec 19;18:977. doi: 10.1186/s12864-017-4318-x (PMC5735816; doi:10.1186/s12864-017-4318-x)
Supplement: Additional file 1: — Collection of all supplementary figures and tables. (DOCX 721 kb) [file 12864_2017_4318_MOESM1_ESM.docx]

**SUPPLEMENTARY FIGURES**

**Figure S1. Quality control statistics in control regions.** Pearson correlation coefficient between normalized read depth values in neighboring windows and Kolmogorov-Smirnov statistic for goodness of normal distribution fit. All samples are autocorrelated with correlation coefficient between 0.08 and 0.12, while this coefficient for bxr samples is ~3 times higher. For all the samples normal distribution is approximates the data, but the bxr sample is the least well approximated.


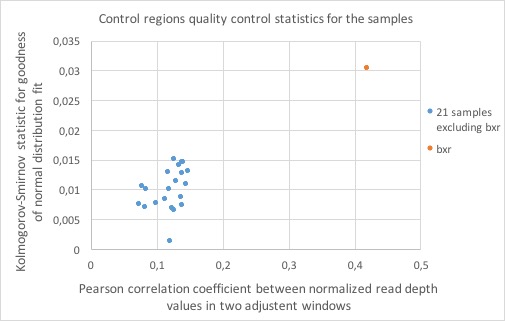


**Figure S2. Length of duplicated sequence on autosomes per sample and coverage.** Total length of duplications called per samples does not depend on the sample coverage.


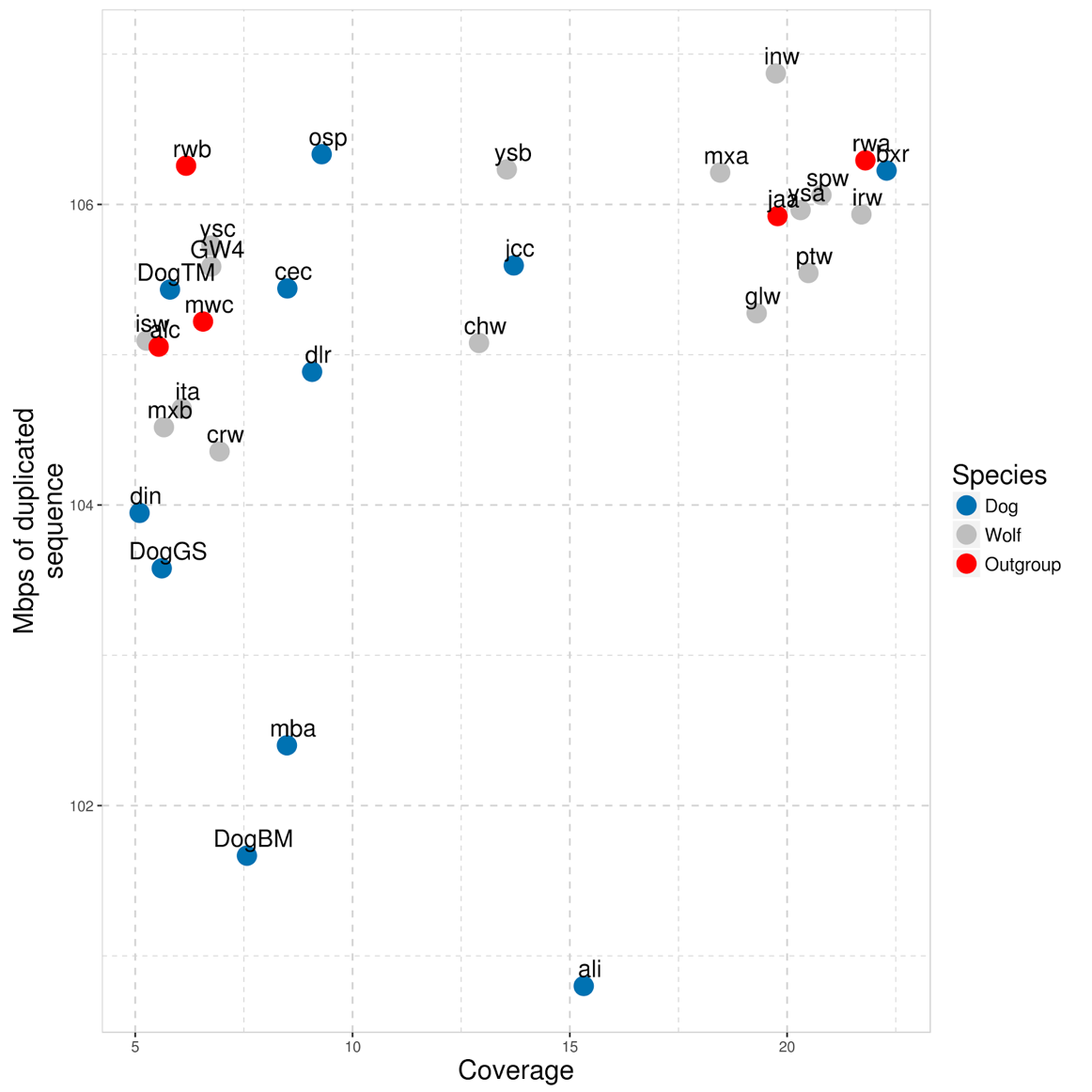


**Figure S3.  Significant overlap between canine SDs and genes.** Distribution of the number of transcripts overlapping with SDs shared across all thirty-two samples, after shuffling the genomic coordinates of duplications 10,000 times, compared with the true overlap observed (red line). The randomization p-value was calculated as the proportion of permutations in which a value greater than the true value was obtained.


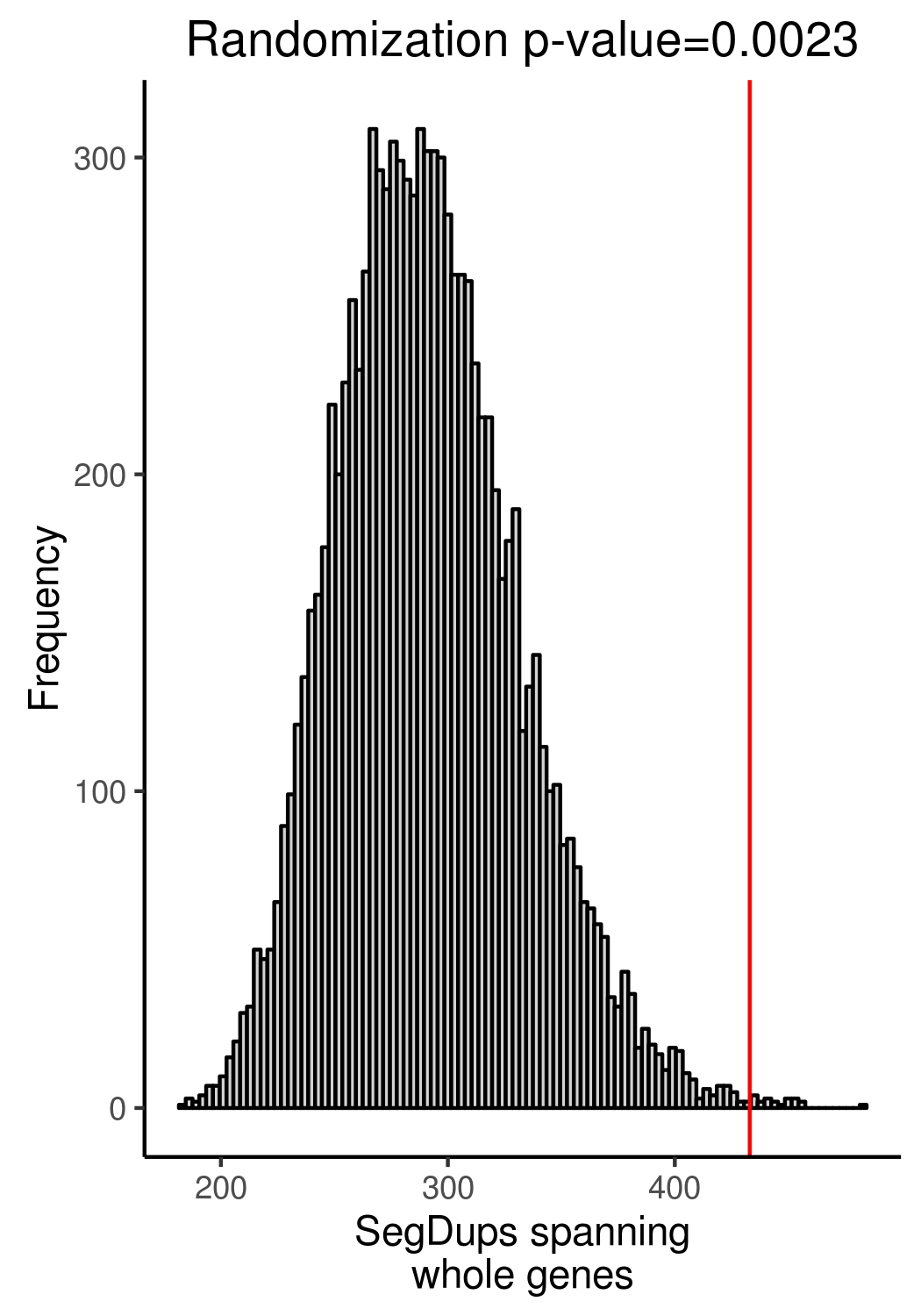


**Figure S4. Sharing of dog and gray wolf specific duplicated windows within individuals of the same species.** On each bar we show the percentage of duplicated sequence relative to the total length of duplications we identified exclusively in dogs (1.36 Mbps) or gray wolves (0.63 Mbps). These numbers correspond to the fraction of the private duplication track which is not bound by consecutiveness and thus is strictly duplicated in one species and non-duplicated in the other.


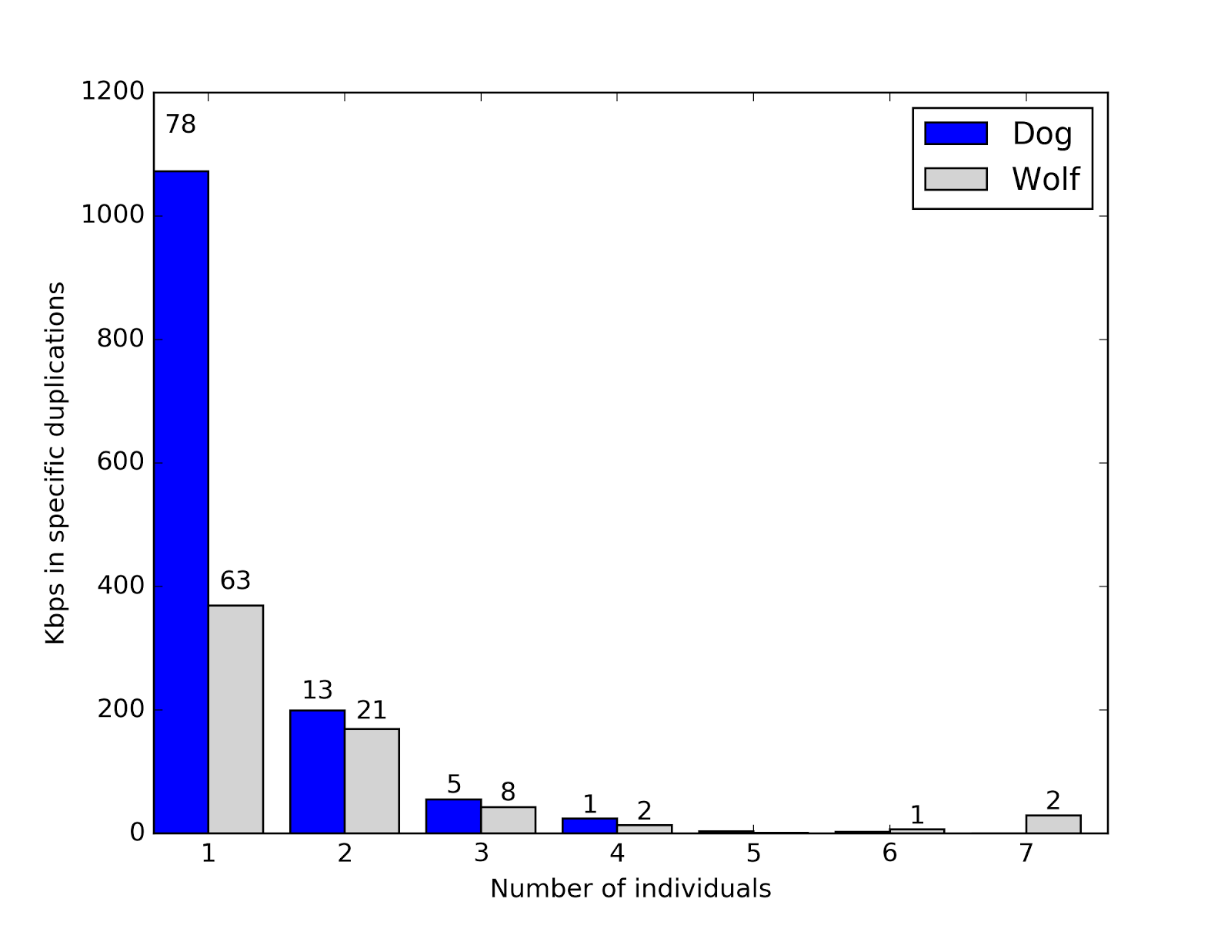


**Figure S5. Overlap between diverse windows in dogs and wolves.** Percentage indicates the proportion of diverse windows which are not found diverse in the other species.

**Figure S6. Accuracy and coverage. (A)** aCGH validation rates per sample as percentage of predicted gains in copy number compared to boxer, which are validated with aCGH (median aCGH value is above determined threshold); **(B)** Pearson correlation coefficient between experimental aCGH signal and digital dCGH value of log2 ratio of predicted copy number in the sample to predicted copy number in bxr.

**Figure S7. Proportion of genomic duplications which are variable, controlling for number of copies in the reference genome.**


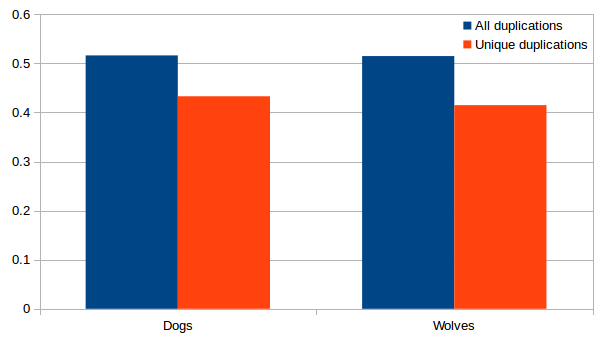


**Figure S8. aCGH signal distribution.** (A) Histogram of the mean diploid aCGH signal for all samples. Vertical lines correspond to the chosen thresholds. Green: distribution of sample medians. Blue: distribution of sample means; (B) Density plot of aCGH signal values for all samples. Sample ysc (colored in red) stands out as an outlier. (C) Per sample correspondence of aCGH values (gains in green and losses in red) with predicted CN intervals (in grey). Concatenated genomic regions which experience CN event on the X axis and log_2_ CN ratio on the Y axis.


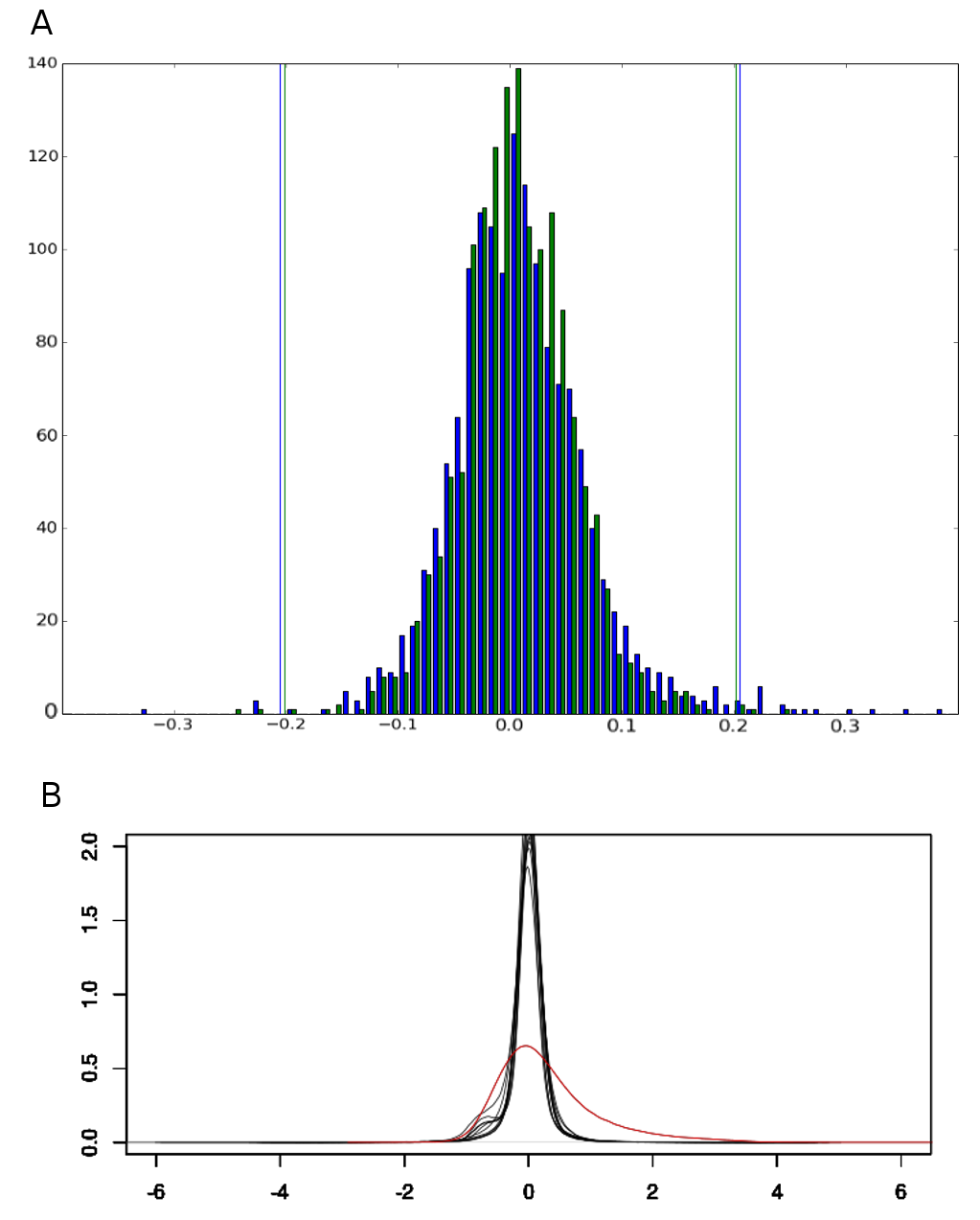


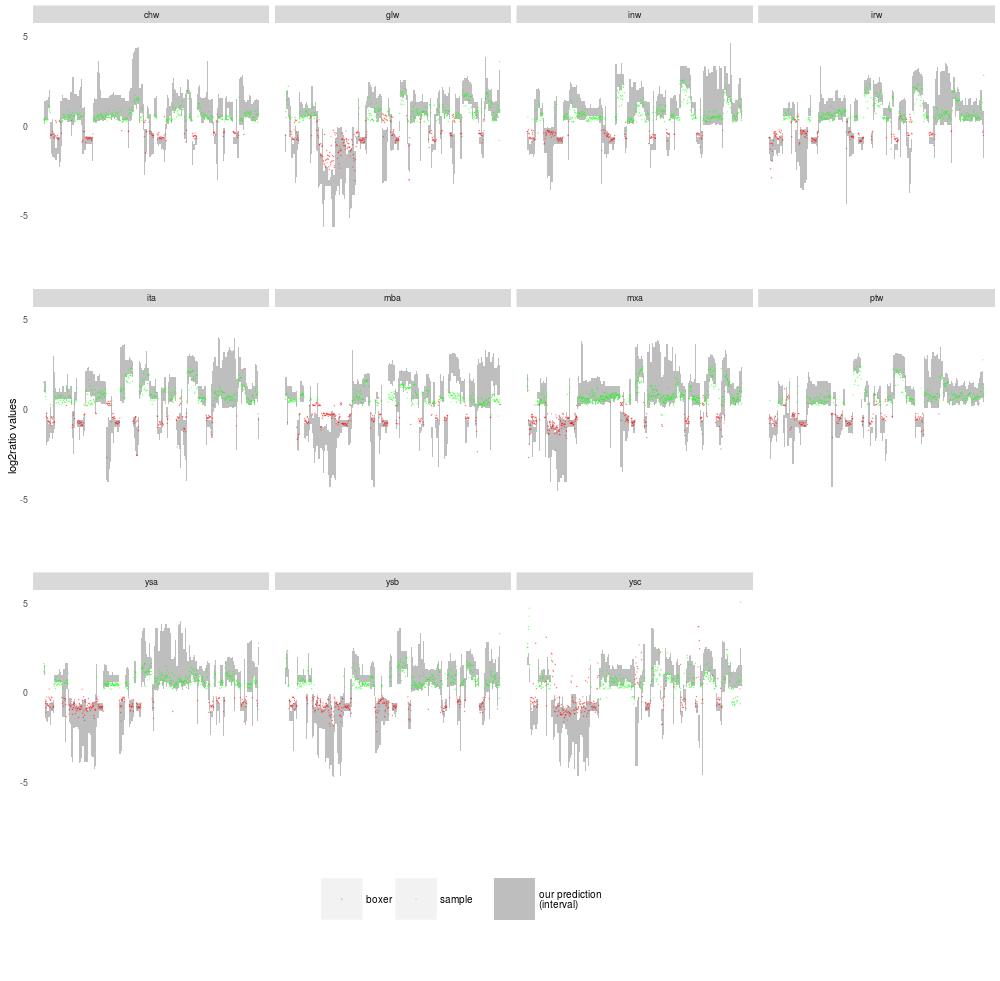
C

**SUPPLEMENTARY TABLES**

**Table S1. Comparison of copy number predictions with aCGH data.** For each of the 14 samples we calculated the Pearson correlation coefficient between sequencing-based "digital" and aCGH log2ratios in windows inside genomic duplications with 2 or more aCGH probes. Additionally, for each sample we calculated the percentage of validated duplicated windows, which passed the aCGH_CUTOFF_=±0.2.

|  | Gain relative to reference | | | Loss relative to reference | | | R, Correlation Coefficient |
| --- | --- | --- | --- | --- | --- | --- | --- |
| Sample | Validated | Total | % Validated | Validated | Total | % Validated |  |
| chw | 93 | 101 | 92.1 | 84 | 124 | 67.7 | 0.70 |
| glw | 248 | 256 | 96.9 | 111 | 157 | 70.7 | 0.83 |
| inw | 264 | 277 | 95.3 | 78 | 111 | 70.3 | 0.80 |
| irw | 243 | 246 | 98.8 | 100 | 168 | 59.5 | 0.78 |
| ita | 274 | 280 | 97.9 | 89 | 124 | 71.8 | 0.79 |
| mba | 180 | 204 | 88.2 | 96 | 155 | 61.9 | 0.62 |
| mxa | 223 | 234 | 95.3 | 102 | 144 | 70.8 | 0.79 |
| ptw | 151 | 152 | 99.3 | 92 | 148 | 62.2 | 0.79 |
| ysa | 275 | 288 | 95.5 | 107 | 140 | 76.4 | 0.80 |
| ysb | 257 | 272 | 94.5 | 102 | 125 | 81.6 | 0.80 |
| **Average** | **95.4** | | | **69.3** | | | **0.77** |
| **SD** | **3.3** | | | **6.8** | | | **0.06** |

**Table S2. Functional enrichment analysis of duplicated genes.** Terms have been sorted by dispensability as a summary for both relevance and singularity. Frequency: relative frequency of a term in the whole GO term database. Annotated: Total number of genes annotated to a term. Significant: Number of genes annotated to a term which are completely spanned by segmental duplications. Dispensability: Measures how redundant a term is when compared semantically to its GO cluster counterparts. Uniqueness: Measures how much of an outlier a term is when semantically compared to the whole list of GO terms. Conditional p-value: Conditional p-value of a GO term calculated via the elimination algorithm of the TopGO package accounting for GO topology.

| **Term ID** | **Description** | **Frequency** | **Annotated** | **Significant** | **Dispensability** | **Uniqueness** | **Conditional p-value** |
| --- | --- | --- | --- | --- | --- | --- | --- |
| GO:0050911 | detection of chemical stimulus involved in sensory perception of smell | 0.09% | 533 | 146 | 0 | 0.706 | 0.0000 |
| GO:0002377 | immunoglobulin production | 0.01% | 82 | 21 | 0 | 0.886 | 0.0000 |
| GO:0006910 | phagocytosis, recognition | 0.00% | 20 | 7 | 0.03 | 0.903 | 0.0000 |
| GO:0090267 | positive regulation of mitotic cell cycle spindle assembly checkpoint | 0.00% | 7 | 2 | 0.041 | 0.556 | 0.0089 |
| GO:0071615 | oxidative deethylation | 0.00% | 1 | 1 | 0.042 | 0.944 | 0.0214 |
| GO:0016098 | monoterpenoid metabolic process | 0.00% | 2 | 1 | 0.044 | 0.911 | 0.0424 |
| GO:0006768 | biotin metabolic process | 0.14% | 1 | 1 | 0.06 | 0.848 | 0.0214 |
| GO:0036065 | fucosylation | 0.02% | 12 | 2 | 0.074 | 0.923 | 0.0262 |
| GO:0009403 | toxin biosynthetic process | 0.04% | 1 | 1 | 0.078 | 0.874 | 0.0214 |
| GO:0018894 | dibenzo-p-dioxin metabolic process | 0.00% | 1 | 1 | 0.12 | 0.908 | 0.0214 |
| GO:0009820 | alkaloid metabolic process | 0.00% | 1 | 1 | 0.149 | 0.944 | 0.0214 |
| GO:1903826 | arginine transmembrane transport | 0.14% | 7 | 3 | 0.196 | 0.858 | 0.0003 |
| GO:0006953 | acute-phase response | 0.00% | 12 | 3 | 0.249 | 0.883 | 0.0019 |
| GO:0007186 | G-protein coupled receptor signaling pathway | 0.37% | 1072 | 147 | 0.35 | 0.725 | 0.0000 |
| GO:0042738 | exogenous drug catabolic process | 0.00% | 2 | 1 | 0.41 | 0.848 | 0.0424 |
| GO:0060309 | elastin catabolic process | 0.00% | 1 | 1 | 0.414 | 0.899 | 0.0214 |
| GO:0006958 | complement activation, classical pathway | 0.00% | 12 | 7 | 0.467 | 0.668 | 0.0000 |
| GO:0050918 | positive chemotaxis | 0.00% | 25 | 3 | 0.468 | 0.852 | 0.0158 |
| GO:0051541 | elastin metabolic process | 0.00% | 2 | 1 | 0.509 | 0.901 | 0.0424 |
| GO:0050871 | positive regulation of B cell activation | 0.00% | 57 | 7 | 0.542 | 0.716 | 0.0002 |
| GO:0042742 | defense response to bacterium | 0.08% | 115 | 8 | 0.574 | 0.833 | 0.0033 |
| GO:0016579 | protein deubiquitination | 0.01% | 85 | 5 | 0.607 | 0.867 | 0.0357 |
| GO:0050853 | B cell receptor signaling pathway | 0.00% | 40 | 7 | 0.624 | 0.669 | 0.0000 |
| GO:0044778 | meiotic DNA integrity checkpoint | 0.00% | 1 | 1 | 0.643 | 0.884 | 0.0214 |
| GO:0006955 | immune response | 0.38% | 707 | 33 | 0.652 | 0.769 | 0.0017 |

**Table S3. Dog and gray wolf samples used for the analysis of CNV diversity.** We performed the analysis of CNV variance in a matched group of 11 dogs and 11 gray wolves. We then repeated the analysis excluding the boxer (bxr), which validation rate is lower due to sequencing biases. To match the sample size in dogs and gray wolves, we then excluded the Israeli wolf sample (irw), based on the fact that it was the gray wolf with the lowest raw coverage out of the 11 gray wolves used initially.

| **Species** | **Sample** | **Included** | **Reason for exclusion** |
| --- | --- | --- | --- |
| Dog | ali | Yes |  |
| Dog | bxr | Yes |  |
| Dog | cec | Yes |  |
| Dog | din | Yes |  |
| Dog | dlr | Yes |  |
| Dog | DogCI3 | No | Training sample |
| Dog | DogBM | Yes |  |
| Dog | DogGS | Yes |  |
| Dog | DogTM | Yes |  |
| Dog | jcc | Yes |  |
| Dog | mba | Yes |  |
| Dog | osp | Yes |  |
| Wolf | chw | Yes |  |
| Wolf | crw | Yes |  |
| Wolf | glw | Yes |  |
| Wolf | GW3 | No | Training sample |
| Wolf | GW4 | No | chw is also from China and has a higher raw coverage |
| Gray wolf | inw | Yes |  |
| Gray wolf | irw | Yes |  |
| Gray wolf | isw | Yes |  |
| Gray wolf | ita | Yes |  |
| Gray wolf | mxa | Yes |  |
| Gray wolf | mxb | No | mxa is also from Mexico and has a higher raw coverage |
| Gray wolf | ptw | Yes |  |
| Gray wolf | spw | Yes |  |
| Gray wolf | ysa | Yes |  |
| Gray wolf | ysb | No | ysa is also from Yellowstone and has a higher raw coverage |
| Gray wolf | ysc | No | Son of the Yellowstone trio |

**Table S4. Functional enrichment analysis of private duplicated segments.** Terms have been sorted by dispensability as a summary for both relevance and singularity. Frequency: relative frequency of a term in the whole GO term database. Annotated: Total number of genes annotated to a term. Significant: Number of genes annotated to a term which are completely spanned by segmental duplications. Dispensability: Measures how redundant a term is when compared semantically to its GO cluster counterparts. Uniqueness: Measures how much of an outlier a term is when semantically compared to the whole list of GO terms. Conditional p-value: Conditional p-value of a GO term calculated via the elimination algorithm of the TopGO package accounting for GO topology. (A) Dogs. (B) Wolves

(A)

| **Term ID** | **Description** | **Frequency** | **Annotated** | **Significant** | **Dispensability** | **Uniqueness** | **Conditional p-value** |
| --- | --- | --- | --- | --- | --- | --- | --- |
| GO:0050911 | detection of chemical stimulus involved in sensory perception of smell | 0.086% | 533 | 6 | 0 | 0.554 | 0.0000 |
| GO:0060309 | elastin catabolic process | 0.000% | 1 | 1 | 0 | 0.854 | 0.0012 |
| GO:0002474 | antigen processing and presentation of peptide antigen via MHC class I | 0.060% | 21 | 1 | 0 | 0.906 | 0.0254 |
| GO:0007157 | heterophilic cell-cell adhesion via plasma membrane cell adhesion molecules | 0.002% | 33 | 1 | 0 | 0.913 | 0.0397 |
| GO:0036065 | fucosylation | 0.018% | 12 | 1 | 0.036 | 0.813 | 0.0146 |
| GO:0006826 | iron ion transport | 0.159% | 29 | 2 | 0.042 | 0.909 | 0.0006 |
| GO:0006879 | cellular iron ion homeostasis | 0.130% | 35 | 2 | 0.043 | 0.828 | 0.0008 |
| GO:0006048 | UDP-N-acetylglucosamine biosynthetic process | 0.026% | 4 | 1 | 0.165 | 0.808 | 0.0049 |
| GO:0007186 | G-protein coupled receptor signaling pathway | 0.368% | 1072 | 6 | 0.35 | 0.683 | 0.0012 |
| GO:0006298 | mismatch repair | 0.135% | 19 | 1 | 0.425 | 0.654 | 0.0230 |
| GO:0031017 | exocrine pancreas development | 0.001% | 4 | 1 | 0.519 | 0.67 | 0.0049 |

(B)

| **Term ID** | **Description** | **Frequency** | **Annotated** | **Significant** | **Dispensability** | **Uniqueness** | **Conditional p-value** |
| --- | --- | --- | --- | --- | --- | --- | --- |
| GO:0050911 | detection of chemical stimulus involved in sensory perception of smell | 0.086% | 533 | 13 | 0 | 0.807 | 0.0000 |
| GO:0035563 | positive regulation of chromatin binding | 0.000% | 3 | 1 | 0 | 0.852 | 0.0055 |
| GO:0048007 | antigen processing and presentation, exogenous lipid antigen via MHC class Ib | 0.000% | 8 | 1 | 0 | 0.864 | 0.0146 |
| GO:1903826 | arginine transmembrane transport | 0.140% | 7 | 2 | 0.042 | 0.85 | 0.0001 |
| GO:1900113 | negative regulation of histone H3-K9 trimethylation | 0.000% | 6 | 1 | 0.116 | 0.44 | 0.0110 |
| GO:2001034 | positive regulation of double-strand break repair via nonhomologous end joining | 0.000% | 3 | 1 | 0.208 | 0.724 | 0.0055 |
| GO:0008214 | protein dealkylation | 0.004% | 18 | 1 | 0.276 | 0.763 | 0.0326 |
| GO:0072661 | protein targeting to plasma membrane | 0.001% | 12 | 1 | 0.305 | 0.704 | 0.0218 |
| GO:0007186 | G-protein coupled receptor signaling pathway | 0.368% | 1072 | 13 | 0.35 | 0.683 | 0.0000 |
| GO:0001955 | blood vessel maturation | 0.001% | 5 | 1 | 0.504 | 0.89 | 0.0092 |
| GO:0002475 | antigen processing and presentation via MHC class Ib | 0.000% | 15 | 1 | 0.517 | 0.867 | 0.0272 |
| GO:0060216 | definitive hemopoiesis | 0.002% | 12 | 1 | 0.533 | 0.796 | 0.0218 |
| GO:0033169 | histone H3-K9 demethylation | 0.001% | 7 | 1 | 0.547 | 0.542 | 0.0128 |

**Table S5. Summary of SNP calling.** For the 11 dogs and 11 gray wolves used to compare CNV and SNP diversity, as well as for a subset of these with raw coverage >7X, we show the length of the callable genome after applying several filters, the total number of variant sites resulting from the SNP calling and the number of segregating sites.

|  | **11 dogs + 11 gray wolves** | | **8 dogs + 8 gray wolves**  **(raw coverage >7X)** | |
| --- | --- | --- | --- | --- |
| ***Callable genome (bps)*** |  | |  | |
| Shared across all samples | 792,021,800 | | 1,708,764,705 | |
| Masked regions subtracted | 418,814,869 | | 876,166,428 | |
| Copy number variable 1-Kbps windows subtracted | 394,763,599 | | 836,214,497 | |
| chrX and chrM subtracted  (Final callable genome) | 391,762,228 | | 818,361,274 | |
|  |  | |  | |
| ***Total number of SNPs*** |  | |  | |
| Whole-genome | 19,801,894 | | 19,801,894 | |
| Within genome callable | 2,897,093 | | 6,285,868 | |
| Overall number of segregating sites per bps of callable | 7.40E-03 | | 7.68E-03 | |
|  |  | |  | |
| ***% Segregating sites per bps of callable*** | **Dogs** | **Gray wolves** | **Dogs** | **Gray wolves** |
| 0 missing alleles per species | 4.07 x10^-3^ | 6.43 x10^-3^ | 3.65 x10^-3^ | 6.12 x10^-3^ |
| ≤2 missing alleles per species | 4.07 x10^-3^ | 6.43 x10^-3^ | 3.65 x10^-3^ | 6.12 x10^-3^ |

**Table S6. CNV and SNP diversity between dogs and gray wolves.** The range, standard deviation (std) and standard error of the mean (sem) for the 5,000 bootstrap values are shown. **(A)** SNP diversity was measured as the number of segregating sites in each species relative to the size of the callable genome; **(B)** CNV diversity was measured as the percentage of diverse windows among all windows in duplicated regions. All duplications were split into three categories of low (2-4 mean copy number), medium (5-15 mean copy number) and high copy number (16+ mean copy number). Ratio of percentage of diverse windows in dogs to diverse windows in wolves is shown per category. The data were further filtered to exclude short diverse regions (<5 consecutive diverse windows) and singletons. Analysis was repeated excluding bxr sample and chw (to match the number of individuals).

**A**

| SNP diversity | | | | |
| --- | --- | --- | --- | --- |
|  | Observed | Bootstrap range | std | sem |
| Dogs | 0.00407 | (0.00395, 0.00420) | 0.00006416821 | 0.0000009074755286 |
| Wolves | 0.00643 | (0.00628, 0.00659) | 0.00007982707 | 0.00000112892525 |

**B**

| CNV diversity | | | | | | |
| --- | --- | --- | --- | --- | --- | --- |
|  | | Category | Observed | Bootstrap range | std | sem |
| **All 11 samples** | Overall Diversity | Dogs | 54.50707 | (53.74197, 55.26590) | 0.39301 | 0.00556 |
|  |  | Wolves | 54.62957 | (53.83738, 55.41543) | 0.39865 | 0.00564 |
|  | 1ind 1kbps (Dog-Wolf ratio) | 2-4 | 1.37186 | (1.24579, 1.51957) | 0.06933 | 0.00098 |
|  |  | 5-15 | 0.96336 | (0.93178, 0.99547) | 0.01621 | 0.00023 |
|  |  | 16++ | 1.03861 | (0.99203, 1.08854) | 0.02465 | 0.00035 |
|  | 1ind 5kbps | 2-4 | 1.52786 | (1.34286, 1.73937) | 0.10163 | 0.00144 |
|  |  | 5-15 | 0.94217 | (0.90560, 0.97920) | 0.01887 | 0.00027 |
|  |  | 16++ | 1.04565 | (0.99347, 1.10109) | 0.02705 | 0.00038 |
|  | 2ind 1kbps | 2-4 | 1.18780 | (0.97680, 1.46012) | 0.12216 | 0.00173 |
|  |  | 5-15 | 0.88979 | (0.85386, 0.92671) | 0.01870 | 0.00026 |
|  |  | 16++ | 0.97325 | (0.92543, 1.02540) | 0.02537 | 0.00036 |
| **Without bxr & chw** | Overall Diversity | Dogs | 49.10348 | (48.36898, 49.87455) | 0.38580 | 0.00546 |
|  |  | Wolves | 49.26801 | (48.46948, 50.06021) | 0.39970 | 0.00565 |
|  | 1ind 1kbps | 2-4 | 0.99872 | (1.01523, 1.25739) | 0.06143 | 0.00087 |
|  |  | 5-15 | 0.96904 | (0.94779, 1.01887) | 0.01815 | 0.00026 |
|  |  | 16++ | 1.04347 | (1.01944, 1.12137) | 0.02600 | 0.00037 |
|  | 1ind 5kbps | 2-4 | 1.33424 | (1.16863, 1.52913) | 0.09203 | 0.00130 |
|  |  | 5-15 | 1.00101 | (0.95977, 1.04397) | 0.02154 | 0.00030 |
|  |  | 16++ | 1.08151 | (1.02657, 1.14056) | 0.02854 | 0.00040 |
|  | 2ind 1kbps | 2-4 | 1.20632 | (0.99164, 1.48617) | 0.12595 | 0.00178 |
|  |  | 5-15 | 0.93986 | (0.90113, 0.98016) | 0.02030 | 0.00029 |
|  |  | 16++ | 0.98439 | (0.93515, 1.03829) | 0.02578 | 0.00036 |

**Table S7. aCGH validation rates of diverse windows.** For each sample we calculated the median and mean percentage of validated diverse windows, which passed the aCGH_CUTOFF_=±3*σ_aCGH_(CR).

|  | Loss Mean | Loss Median | Gain Mean | Gain Median |
| --- | --- | --- | --- | --- |
| сhw | 0,94 | 0,94 | 0,83 | 0,89 |
| glw | 0,90 | 0,91 | 0,88 | 0,95 |
| inw | 0,85 | 0,83 | 0,87 | 0,91 |
| irw | 0,72 | 0,76 | 0,90 | 0,93 |
| ita | 0,90 | 0,89 | 0,85 | 0,87 |
| mba | 0,66 | 0,67 | 0,85 | 0,84 |
| mxa | 0,89 | 0,90 | 0,83 | 0,88 |
| ptw | 0,77 | 0,74 | 0,83 | 0,84 |
| ysa | 0,93 | 0,94 | 0,85 | 0,92 |
| ysb | 0,93 | 0,95 | 0,81 | 0,83 |
| All samples | 0,85 | 0,89 | 0,85 | 0,88 |

**Table S8. Functional enrichment analysis of genes in the diverse duplications of low and medium copy number.** Terms have been sorted by dispensability as a summary for both relevance and singularity. Frequency: relative frequency of a term in the whole GO term database. Annotated: Total number of genes annotated to a term. Significant: Number of genes annotated to a term which are completely spanned by segmental duplications. Dispensability: Measures how redundant a term is when compared semantically to its GO cluster counterparts. Uniqueness: Measures how much of an outlier a term is when semantically compared to the whole list of GO terms. Conditional p-value: Conditional p-value of a GO term calculated via the elimination algorithm of the TopGO package accounting for GO topology. (A) Dogs. (B) Wolves.

(A)

| **Term ID** | **Description** | **Frequency** | **Significant** | **Annotated** | **Dispensability** | **Uniqueness** | **Conditional p-value** |
| --- | --- | --- | --- | --- | --- | --- | --- |
| GO:0050911 | detection of chemical stimulus involved in sensory perception of smell | 0.086% | 37 | 535 | 0.00 | 0.60 | 0.00 |
| GO:0002377 | immunoglobulin production | 0.005% | 8 | 82 | 0.00 | 0.90 | 0.00 |
| GO:0006910 | phagocytosis, recognition | 0.001% | 3 | 19 | 0.03 | 0.89 | 0.00 |
| GO:0090267 | positive regulation of mitotic cell cycle spindle assembly checkpoint | 0.000% | 2 | 8 | 0.04 | 0.77 | 0.00 |
| GO:0046041 | ITP metabolic process | 0.001% | 1 | 5 | 0.05 | 0.79 | 0.03 |
| GO:0006310 | DNA recombination | 1.840% | 3 | 136 | 0.11 | 0.76 | 0.04 |
| GO:0009133 | nucleoside diphosphate biosynthetic process | 0.041% | 1 | 9 | 0.21 | 0.64 | 0.05 |
| GO:0046033 | AMP metabolic process | 0.132% | 1 | 12 | 0.29 | 0.71 | 0.06 |
| GO:0042742 | defense response to bacterium | 0.075% | 3 | 121 | 0.31 | 0.67 | 0.03 |
| GO:0007186 | G-protein coupled receptor signaling pathway | 0.368% | 37 | 1081 | 0.35 | 0.64 | 0.00 |
| GO:0060309 | elastin catabolic process | 0.000% | 1 | 1 | 0.41 | 0.87 | 0.01 |
| GO:0006958 | complement activation, classical pathway | 0.001% | 3 | 12 | 0.47 | 0.68 | 0.00 |
| GO:0031017 | exocrine pancreas development | 0.001% | 1 | 4 | 0.52 | 0.79 | 0.02 |
| GO:0050871 | positive regulation of B cell activation | 0.004% | 3 | 56 | 0.54 | 0.79 | 0.00 |
| GO:0006334 | nucleosome assembly | 0.020% | 3 | 81 | 0.54 | 0.93 | 0.01 |
| GO:0006953 | acute-phase response | 0.002% | 1 | 13 | 0.57 | 0.75 | 0.07 |
| GO:0050853 | B cell receptor signaling pathway | 0.003% | 3 | 39 | 0.62 | 0.66 | 0.00 |
| GO:0006955 | immune response | 0.380% | 11 | 692 | 0.65 | 0.70 | 0.02 |
| GO:0006172 | ADP biosynthetic process | 0.002% | 1 | 7 | 0.69 | 0.60 | 0.04 |

(B)

| **Term ID** | **Description** | **Frequency** | **Significant** | **Annotated** | **Dispensability** | **Uniqueness** | **Conditional p-value** |
| --- | --- | --- | --- | --- | --- | --- | --- |
| GO:0050911 | detection of chemical stimulus involved in sensory perception of smell | 0.086% | 40 | 535 | 0.00 | 0.77 | 0.00 |
| GO:0035563 | positive regulation of chromatin binding | 0.000% | 1 | 4 | 0.00 | 0.86 | 0.02 |
| GO:0000478 | endonucleolytic cleavage involved in rRNA processing | 0.001% | 1 | 12 | 0.00 | 0.69 | 0.06 |
| GO:0006910 | phagocytosis, recognition | 0.001% | 3 | 19 | 0.03 | 0.92 | 0.00 |
| GO:0036065 | fucosylation | 0.018% | 1 | 14 | 0.04 | 0.91 | 0.07 |
| GO:0006958 | complement activation, classical pathway | 0.001% | 3 | 12 | 0.24 | 0.63 | 0.00 |
| GO:0042742 | defense response to bacterium | 0.075% | 3 | 121 | 0.31 | 0.71 | 0.02 |
| GO:0007186 | G-protein coupled receptor signaling pathway | 0.368% | 40 | 1081 | 0.35 | 0.65 | 0.00 |
| GO:0090267 | positive regulation of mitotic cell cycle spindle assembly checkpoint | 0.000% | 2 | 8 | 0.41 | 0.45 | 0.00 |
| GO:2001034 | positive regulation of double-strand break repair via nonhomologous end joining | 0.000% | 1 | 2 | 0.44 | 0.61 | 0.01 |
| GO:0033169 | histone H3-K9 demethylation | 0.001% | 1 | 9 | 0.46 | 0.52 | 0.04 |
| GO:0002377 | immunoglobulin production | 0.005% | 5 | 82 | 0.47 | 0.92 | 0.00 |
| GO:0050871 | positive regulation of B cell activation | 0.004% | 3 | 56 | 0.54 | 0.76 | 0.00 |
| GO:0006953 | acute-phase response | 0.002% | 1 | 13 | 0.57 | 0.77 | 0.06 |
| GO:0050853 | B cell receptor signaling pathway | 0.003% | 3 | 39 | 0.62 | 0.65 | 0.00 |
| GO:0006334 | nucleosome assembly | 0.020% | 2 | 81 | 0.66 | 0.54 | 0.06 |

**Table S9. Genes showing greatest copy number expansion in dogs compared to wolves.**

| **Gene** | **Gene name** | **Interpro description** | **Chr** | **Start** | **End** | **Length(bps)** | **Dog** | **Wolf** | **Vst** |
| --- | --- | --- | --- | --- | --- | --- | --- | --- | --- |
| ENSCAFG00000028653 |  | A-amylase_b_C | chr6 | 46954282 | 46955934 | 1652 | 9.5 | 2 | 0.54132 |
| ENSCAFG00000030746 |  |  | chr1 | 60586581 | 60598997 | 12416 | 12 | 2 | 0.18993 |
| ENSCAFG00000006763 |  | Ig_C1-set | chr24 | 19240284 | 19249801 | 9517 | 5 | 2.5 | 0.41746 |
| ENSCAFG00000029857 |  |  | chr10 | 20640936 | 20642272 | 1336 | 4 | 2.5 | 0.15539 |
| ENSCAFG00000029259 | TBPL1 | TBP | chr9 | 29270727 | 29272667 | 1940 | 3.5 | 2.5 | 0.23536 |
| ENSCAFG00000009988 |  | Ribosomal_L37ae | chr28 | 14547564 | 14547780 | 216 | 3.5 | 2.5 | 0.20661 |
| ENSCAFG00000001451 |  | DUF4534 | chr12 | 6243548 | 6244100 | 552 | 3.5 | 2.5 | 0.17963 |
| ENSCAFG00000017857 |  | HAX-1 | chr5 | 36398598 | 36399436 | 838 | 3.5 | 2.5 | 0.38673 |
| ENSCAFG00000010808 | OR4L1 | GPCR_Rhodpsn | chr8 | 795895 | 796834 | 939 | 3.5 | 2.5 | 0.28355 |
| ENSCAFG00000009406 |  | Cyclin_C-dom | chr32 | 9993325 | 9996947 | 3622 | 3 | 2.5 | 0.43630 |
| ENSCAFG00000001921 |  | DNA/RNA-bd_Alba-like | chr11 | 51284701 | 51285193 | 492 | 3 | 2.5 | 0.30769 |
| ENSCAFG00000005678 | LGALS7 | Galectin_CRD | chr1 | 114309692 | 114311761 | 2069 | 3 | 2.5 | 0.27522 |
| ENSCAFG00000011435 |  | S10_plectin_N | chr9 | 13211789 | 13212287 | 498 | 3 | 2.5 | 0.19064 |
| ENSCAFG00000023660 |  | DH_sc/Rdtase_SDR | chr31 | 31364085 | 31365294 | 1209 | 3 | 2.5 | 0.16637 |
| ENSCAFG00000032387 | GPR25 | GPCR_Rhodpsn | chr7 | 2304945 | 2305986 | 1041 | 3 | 2.5 | 0.16438 |
